# Assessment of Adherence to Insulin Injections among Diabetic Patients on Basal-Bolus Regimen in Primary and Secondary Healthcare Centers in Al-Jouf Region of Saudi Arabia; A Descriptive Analysis

**DOI:** 10.3390/jcm12103474

**Published:** 2023-05-15

**Authors:** Aseel Awad Alsaidan, Omar Awad Alsaidan, Tauqeer Hussain Mallhi, Yusra Habib Khan, Abdulaziz Ibrahim Alzarea, Abdullah Salah Alanazi

**Affiliations:** 1 Department of Family and Community Medicine, College of Medicine, Jouf University, Sakaka 72388, Al-Jouf Province, Saudi Arabia; 2 Department of Pharmaceutics, College of Pharmacy, Jouf University, Sakaka 72388, Al-Jouf Province, Saudi Arabia; 3 Department of Clinical Pharmacy, College of Pharmacy, Jouf University, Sakaka 72388, Al-Jouf Province, Saudi Arabiaaizarea@ju.edu.sa (A.I.A.);; 4 Health Sciences Research Unit, Jouf University, Sakaka 72388, Al-Jouf Province, Saudi Arabia

**Keywords:** diabetes, adherence, insulin, compliance, complications, medications

## Abstract

Background: Patient adherence to insulin therapy is one of the major challenges during the treatment of diabetes mellitus. Considering the dearth of investigations, this study aimed to determine the adherence pattern and factors linked with nonadherence among diabetic patients using insulin in Al-Jouf region of Saudi Arabia. Methods: This cross-sectional study included diabetic patients using basal-bolus regimens, whether they had type 1 or type 2 diabetes. This study’s objective was determined using a validated data collection form that included sections on demographics, reasons for missed insulin doses, list of barriers to therapy, difficulties during insulin administration, and factors that may improve insulin inaction adherence. Results: Of 415 diabetic patients, 169 (40.7%) were reported to forget doses of insulin every week. The majority of these patients (38.5%) forget one or two doses. Away from home (36,1%), inability to adhere to the diet (24.3%) and embarrassment to administer injections in public (23.7%) were frequently cited as reasons for missing insulin doses. The occurrence of hypoglycemia (31%), weight gain (26%), and needle phobia (22%) were frequently cited as obstacles to insulin injection use. Preparing injections (18.3%), using insulin at bedtime (18.3%), and storing insulin at a cold temperature (18.1%) were the most challenging aspects of insulin use for patients. Reduction in the number of injections (30.8%) and convenient timing for insulin administration (29.6%) were frequently cited as factors that may improve participant adherence. Conclusions: This study revealed that the majority of diabetic patients forget to inject insulin, primarily as a result of travel. By identifying potential obstacles faced by patients, these findings direct health authorities to design and implement initiatives to increase insulin adherence among patients.

## 1. Introduction

Diabetes mellitus (DM) is a disease in which the body’s cells can no longer utilize the insulin produced by the pancreas, or the pancreas can no longer produce enough insulin [1]. DM is a long-lasting, aggressive disorder that eventually leads to losing one or more of the body’s organs [2]. During the last three decades, DM has become one of the top health issues [3], with more than 422 million people currently living with this disorder, and it is estimated to affect more than 640 million people around the world in 2040 [4].

Patient failure to comply with a healthy lifestyle or glucose-lowering medications leads to fatal diabetes mellitus complications [5]. According to the American Diabetes Association (ADA), several factors, such as patient, medication, and system factors, can affect patients’ adherence to diabetes mellitus medications [6]. Insulin therapy is the mainstay of treatment for type 1 (T1DM) and type 2 (T2DM) diabetes mellitus patients [7]. The existing evidence suggests that early initiation of insulin therapy results in a decreased rate of complications and preservation of beta cell function among patients with T2DM [8]. However, patient perceptions and misconceptions regarding the insulin use are significant barriers to insulin therapy [9].

Adherence to insulin therapy is a critical factor for the control of diabetes [10]. Several studies from Saudi Arabia have enumerated various factors affecting adherence to the treatment of DM, including male gender, fear of injection, fear of hypoglycemia, injection site reactions, and poor self-administration technique of insulin due to the lack of adequate knowledge [11,12]. A cross-sectional survey on adherence to the insulin injection showed that only 61.9% of the patients adhered to the therapy, where a higher level of adherence was observed in young people aged 14 to 29 years [13]. A study from Ethiopia showed that only one-fourth (24.2%) of the participants were adherent to their insulin therapy [10]. A Malaysian study on 249 patients also showed adherence in only 8.4% of the study participants. This study reported various factors of adherence, including self-monitoring of blood glucose (SMBG), exercise, and the number of daily insulin injections [14]. Another study ascertained the adherence of the patient to insulin injections, and found that 33% of the patients were not adherent to their medications. This study reported various factors linked with the adherence, such as feeling better, getting heart disease, and staying out of the home for long time [15]. A recent study from Oman indicated frequent blood glucose checks as a potential barrier of adherence among diabetic patients [16]. A Turkish study showed adherence to insulin treatment in insulin-naïve type 2 diabetic patients at 44.3%. This study also demonstrated that younger patients with a shorter duration of diabetes and antidiabetic treatment are more likely to be nonadherent [17]. There is a dearth of investigations on factors linked with the nonadherence to the basal-bolus insulin regimen in Saudi Arabia, particularly in the northern region of the country. In this context, this study aimed to assess the adherence and its factors among diabetic patients who were using insulin in the Al-Jouf region of Saudi Arabia.

## 2. Materials and Methods

Ethics: The ethical approval for this study was granted by the local committee of bioethics (LCBE) at Jouf University (approval No. 25-10-43).

Study Population and Design: This cross-sectional study was conducted during 1 July to 30 October 2022 in primary and secondary healthcare centers in the Al-Jouf region of Saudi Arabia. This study included diabetic patients, either with type 1 or type 2 diabetes and using basal-bolus regimens, having age more than 14 years, and were self-administering the insulin injections. Diabetic patients who were below 14 years, pregnant, and had mental problems, or with gestational diabetes, were excluded from this study. Patients under the age of 14 are managed by the pediatric endocrinology unit and are therefore excluded from this study.

Study Site: The data were collected from seven primary healthcare centers, three general hospitals, one diabetes center, and three private clinics in the Al-Jouf region of Saudi Arabia.

Sample size and Sampling Technique: We used the expected level of adherence, which was reported as 61% in the given literature, for calculating our sample size. Under simple random sampling with the margin of error at 5% and the confidence level at 95%, we will need a sample of size 415. We use the following formula.

(1) $$n=\frac{z^{2}P\left(1-P\right)}{d^{2}}$$

where, *n* = sample size, *z* = z statistic for the level of confidence, *P* = expected prevalence, and *d* = allowable error. This formula assumes that “*P*” and “*d*” are decimal values. The data were collected through a convenient sampling technique.

Study Instrument: A pre-validated, reliable (Cronbach alpha: 0.833), and self-administered survey tool was used to collect the data from diabetic patients. The study instrument consisted of various sections. Section I contained demographics data (gender, age, educational level), type and duration of diabetes, number of daily insulin injections, presence of other chronic diseases, healthcare facilities for follow-up, and adherence to routine follow-up. Section II had items to identify the reasons for missing insulin doses. Section III consisted of a list of barriers, and the participants were asked to select potential barriers of insulin administration. Section IV had items to identify the potential difficulties during the administration of insulin injection. Section V had items related to the factors that may increase the adherence of insulin injections, and the participants were asked to select the factors according to their understanding. A similar type of questionnaire has been used in the literature [13]. The questionnaire was translated from English to Arabic through a forward-and-backward translation process. The questionnaire was administered in Arabic. An Informed consent was requested for all participants.

Data Collection: There were neither laboratory nor investigational techniques that were applied in this study. The patients, who agreed to participate, were requested to fill in the data collection form. However, the data collection form and the purpose of the study were explained to the participants. The participants were given a considerable amount of time fill out the questionnaire in a comfortable environment. All the data were collected and transferred to a Microsoft Excel spreadsheet for the purpose of cleaning. Subsequently, the data were transferred to SPSS for coding and analysis. Only the investigators of the study had access to the data.

Statistical Analysis: Descriptive statistics were applied through SPSS version 25. Categorical data were presented as numbers along with proportion. Inferential statistics were not applied in this study.

## 3. Results

A total of 415 diabetic patients participated in this study. Most of the participants belonged to age between 30 and 60 years, and there was almost equal distribution of male and female gender (48.4% versus 51.6%). The majority of the patients (49.6%) had type 2 DM (T2DM), while only 21.2% reported to have type 1 DM (T1DM). Unfortunately, 29.2% of the study participants were not sure about the type of DM. More than half of the patients had a diabetes duration of more than 5 years. Twenty-six percent of the patients reported to not have routine follow-up for diabetes care, while approximately half of the participants (47.2%) were seeking follow-up in either secondary or tertiary healthcare facilities. Around 22% of the patients indicated no follow-up for diabetes care in the preceding year. However, 45.1% of the patients did not miss their follow-up during the last year. More than half of the patients (58.8%) reported that their diabetes is controlled. Approximately one-half of the participants had chronic diseases, where hyperlipidemia (20.7%) and hypertension (20%) were more profound (Table 1).

### 3.1. Adherence to Insulin Doses

Most of the participants (*n* = 184, 44.3%) were taking four insulin injections per week, followed by two (*n* = 150, 36.1%) and three (*n* = 81, 19.5%) injections. More than one-third of the study population (*n* = 169, 40.7%) reported to forget doses of insulin every week. Of these participants (*n* = 169), 38.5% (*n* = 65/169) forget 1–2 doses, 33.7% (*n* = 57/169) forget 3–4 doses, and 15.4% (*n* = 26/169) and 12.4% (*n* = 21/169) forget 5–6 doses and >6 doses, respectively. Interestingly, 59.3% (*n* = 246/415) of the overall participants indicated that they do not forget to take insulin doses as per schedule (Table 2). It is important to note that the proportion of forgetting the insulin injections was significantly (*p* < 0.001) higher among patients with T1DM as compared to those with T2DM. Moreover, the frequency of missing doses was also significantly higher among patients with T1DM.

We asked the participants about possible reasons to miss the insulin doses. Being away from home (36.1%), inability to adhere with the diet (24.3%), and embarrassment to take injections in public places (23.7%) were frequently reported reasons in this study (Table 3). Only 39.6% (*n* = 67/169) of the participants reported only one reason for missing insulin doses, while 23.3% (*n* = 90/169) and 7.1% (*n* = 12/169) of the participants reported at least two and three reasons for missing insulin doses, respectively.

We inquired about the handling of missing doses from the study participants. Approximately half of the participants who miss the insulin doses (*n* = 83/169. 49.1%) wait for the next regular dose, while 22.8% (*n* = 90/169) take the dose immediately when they recall, and 22.5% (*n* = 90/169) double the next dose. We also inquired about the practice to check the expiry dates of the insulin injections. Unfortunately, more than half (52.3%) of the total participants do not check expiry dates before insulin administration.

### 3.2. Self-Reported Barriers Experienced by the Patients during Insulin Administration

**Figure 1:**
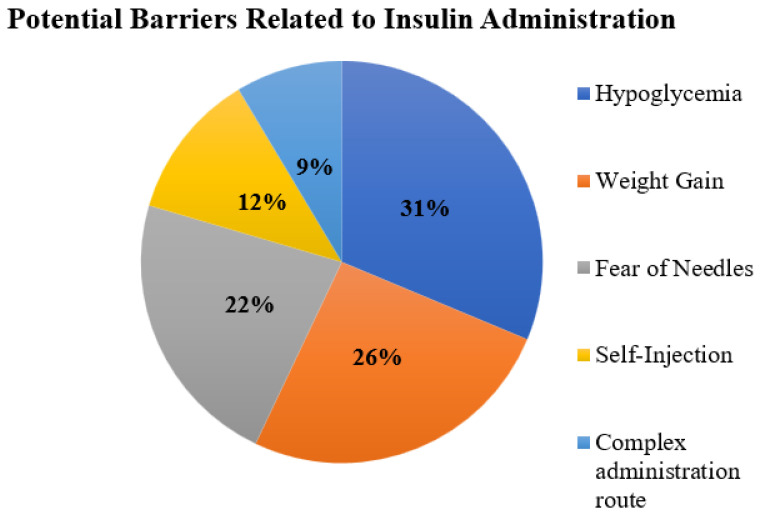
Self-reported difficulties during administration of insulin injections.

We asked about the opinion of the study participants regarding potential barriers associated with insulin use. The occurrence of hypoglycemia (31%), weight gain (26%), and the fear of needles (22%) were commonly reported barriers during the use of insulin injections (Figure 1).

### 3.3. Difficulties among Diabetic Patients during Insulin Use

**Figure 2:**
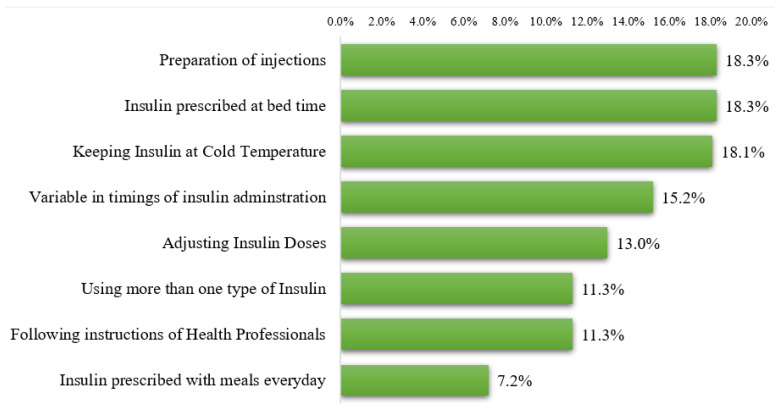
Self-reported difficulties experienced by diabetic patients during insulin use.

We inquired about the difficulties during insulin use among patients. The most common difficulties reported by the study participants were preparation of injections (18.3%), use of insulin at bed time (18.3%), and storage of insulin at a cold temperature (18.1%) (Figure 2).

### 3.4. Self-Reported Factors Improving Adherence to Insulin Injections

A reduction in the number of injections (30.8%) and convenient timing for insulin administration (29.6%) were frequently cited as factors that may improve participant adherence (Table 4). Confidence in taking medications in public was the third most prevalent factor in enhancing adherence.

## 4. Discussion

To the best of our knowledge, this is the first study of its kind to evaluate adherence to insulin injections among diabetic patients residing in the northern region of Saudi Arabia. Our findings indicate that more than one-third of the study population forgets to take insulin doses every week, with traveling away from home being the most common reason, followed by the inability to adhere to a diet, and embarrassment of administering insulin in public places. According to a recent estimate, the burden of diabetes mellitus in Saudi Arabia is expected to increase two times by 2045 [18]. Saudi Arabia ranks seventh globally and second in the middle east for the Diabetes Patient Prevalence Index [19]. The health authorities in the country are struggling to combat the growing encumbrance of the disease amid various challenges. Nonadherence to the therapeutic regimen is one of the major challenges in diabetes control measures [20]. In this context, evaluation of adherence along with factors associated with nonadherence is of paramount importance to direct the policymakers for optimal control of the disease.

Since the burden of diabetes mellitus is linked with its control, which is directly related to adherence, nonadherence should be considered a significant health concern of priority [13]. Our study indicated that 40.7% of diabetic patients forget to take insulin doses every week. These findings are consistent with other studies conducted in Saudi Arabia [5,13,21,22,23]. This study showed adherence to insulin administration at 59.3%, which is closely related to the proportion of adherence reported by Alsayed et al. [13]. Likewise, Almaghaslah et al. indicated 38% nonadherence to insulin regimens, and these results corroborate our findings [23]. The proportion of forgetting insulin doses was significantly higher among patients with T1DM as compared to T2DM. In contrast, the existing literature provides evidence of higher adherence among people with T1DM due to young age and fear of diabetes-related complications in these patients compared to those with T2DM. Since most of the patients in our study were not young, and many patients did not know the type of DM they had, the relationship between the type of DM and insulin forgetfulness cannot be established through our analysis. It is important to note that the prevalence of adherence to insulin therapy widely varies, and ranges from 43% to 86% across the literature [24]. These disparities across the literature are primarily linked with methodological differences, including sample size, type of tool used to measure adherence, and demographic features of the study participants. Taken together, a systematic review of 17 studies indicates that adherence to insulin therapy is generally poor [24], as also shown in our study, which warrants an urgent need for measures to identify factors underlying nonadherence, and to develop strategies to improve adherence to insulin therapy.

Despite advances in drug development and device technology, diabetic patients are still failing to reach glycemic targets. Poor glycemic control is associated with detrimental impacts, such as the increased prevalence of diabetes-related complications, as demonstrated in recent epidemiological studies [25]. Since a considerable proportion of the diabetic patients in our study reported forgetting insulin doses every week, it is imperative to understand the reasons behind such forgetfulness. Our study indicated that the patients’ schedules or daily activities keep them away from their homes, which contributes to forgetfulness. These results are consistent with other studies evaluating the reasons for forgetting the insulin doses [17,26,27]. Being away from home has also recently been discussed as the most common reason to miss insulin doses among diabetic patients [28]. Poor diet adherence was the second common reason for omission/nonadherence to insulin doses in our study, and similar findings have been previously reported [29]. Embarrassment over administering insulin in public places was the third commonly reported reason among the study participants. Being embarrassed or uncomfortable administering doses in public or social situations is considered a major factor linked with suboptimal insulin use [21,26,27,28,30,31,32]. Other factors, such as time constraints, inappropriate timing for insulin administration, the inherent complexity of insulin regimens, and fear of pain and hypoglycemia were observed as reasons to miss the insulin doses. It is important to note that these factors are modifiable and can be controlled through targeted measures. However, communicating these reasons objectively to healthcare professionals is key to addressing them in a timely manner. Our results also showed that most of the patients do not check the expiry of insulin before use, indicating the poor education among them. Given the potential adverse consequences of missing or mistiming insulin doses, healthcare professionals should consider individualized interventions to ensure optimal adherence to insulin therapy among diabetic patients. Diabetes education through interprofessional collaboration is a well-established tool to ensure optimal medication compliance among patients [33,34].

Hypoglycemia, weight gain, and fear of needles were commonly reported barriers in this study. These factors have been indicated by various studies conducted across the globe [24,35,36]. Several other studies from the Middle East indicate that phobia of needles, and adverse events such as weight gain and hypoglycemia, have been well discussed in the literature [16,37]. It is important to understand the inherent nature of these barriers that are linked with insulin use. However, the existing data indicate that inadequate disease and medication knowledge significantly contributes to these barriers among diabetic patients. In this regard, interprofessional collaboration to improve disease awareness and education, and measures to manage adverse events during disease management, appear to be effective.

Preparation, storage, and administration of insulin at bedtime were frequently cited as problematic aspects of insulin use. With an insulin pen, the difficulties associated with preparation can be eliminated. However, insulin administration schedules are sometimes determined by the needs and guideline recommendations of the patient. The timing of insulin injections can be adjusted to the patient’s convenience. Patient preferences and flexible dosing have been discussed in prior studies [38]. It is important to note that the study participants reported reducing the number of injections and flexible timing as factors to improve their adherence to insulin therapy. Evans et al. indicated that flexible dosing and fewer injections have a positive impact on the health-related quality of life among diabetic patients, which potentially may enhance therapy adherence and could contribute to improved long-term outcomes [39]. Similar findings have been observed in another study [40]. The authors of both studies reported a greater impact of the flexibility among people treated with basal-only insulin as compared to those using bolus injections [39,40]. The confidence among patients to take insulin at public places can be boosted though appropriate educational campaigns. Social stigma and embarrassment to administer insulin is a serious issue [41], and should be addressed on a priority basis. Using syringes in a public place may result in feeling socially embarrassed and rejected, leading to feeling that daily insulin injection routines must be hidden from others [42,43]. These perceptions can lead to omissions, delays, or early injections. Allen et al. provided detailed interventions to address the psychological issues among insulin users, including (1) teaching and providing explanations, (2) demonstrations and sharing examples of success using insulin therapy, (3) return demonstrations, and (4) addressing feelings and positively managing expectations [44]. We believe that these interventions would be of great value to improve the adherence among insulin users.

Alarmingly, more than one-quarter of diabetic patients in this study were unaware of their type of diabetes mellitus. These results might relate with the low level of disease knowledge among patients in the region. It is important to note that diabetes education plays a fundamental role in treatment success. Diabetes education empowers the patients to manage their own conditions effectively [45]. Various studies have demonstrated positive impacts of educational programs on outcomes of patients [33,46]. Previous investigations have also indicated unsatisfactory knowledge of diabetes mellitus among the Saudi population [47]. The low level of disease-related knowledge among the study participants can be a possible reason for poor adherence in our study. It can be evident through our results that out of the patients who were not aware of their type of DM, 41.3% forget to take insulin doses. Our findings urge the need for implementation of structured educational courses or programs for diabetic patients.

The findings of this study should be viewed with a few limitations in mind. First, self-reporting approaches may over- or under-estimate adherence. Second, the participant bias, selection bias, and patients’ introspective abilities cannot be disregarded in this study. Thirdly, the data were obtained at a specific period, and adherence may vary if the same group is evaluated at other time points. Fourth, sample convenience may impede the generalizability of the findings. Fifth, the study population was from one of the thirteen regions of Saudi Arabia, and the findings may not be generalizable to other regions. Sixth, the effect of the relationship between patients and healthcare professionals on adherence was not assessed, which has a substantial impact on patient adherence. Last but not least, adherence was tested by a single question relating to forgetting to take insulin every week, and the use of alternative instruments to estimate adherence may lead to inconsistent results. Nevertheless, this study has the major strength of being the first report on insulin adherence among patients residing in northern Saudi Arabia, a region with limited healthcare facilities compared to most other regions across the country. Additionally, our analysis provides valuable insights to the existing literature about adherence among diabetic patients, enabling health authorities to design and implement educational initiatives across the region. This study additionally highlights the importance of educational initiatives, because many patients did not know which type of diabetes mellitus they had.

## 5. Conclusions

According to our analysis, more than one-third of study participants forget to take insulin weekly. Being away from home, inability to adhere to the diet, and embarrassment to administer injections in public were the most frequent causes of dose omission. Approximately half of the participants who miss insulin doses wait for the next scheduled dose. Hypoglycemia, weight gain, and needle phobia were frequently cited as obstacles to insulin injection use. Insulin users frequently reported difficulties with preparing injections, administering insulin at bedtime, and storing insulin at a cold temperature. Patients with diabetes have indicated that reducing the number of injections and administering insulin at convenient times can improve adherence. These findings direct health authorities to design and implement initiatives to increase insulin adherence among patients by identifying potential obstacles faced by patients.

## Figures and Tables

**Table 1:** Demographics of study participants.

Variables	Frequency (*n*)	Proportion (%)
Age		
14–29 Years	84	20.2
30–44 Years	98	23.6
45–60 Years	129	31.1
>60 Years	104	25.1
Gender		
Male	201	48.4
Female	214	51.6
Education Level		
Illiterate—No Formal Education	32	7.7
Primary School	61	14.7
Secondary School	61	14.7
High School	128	30.8
Graduate	104	25.1
Postgraduate	29	7.0
Type of Diabetes Mellitus		
Not Sure	121	29.2
T1DM	88	21.2
T2DM	206	49.6
Duration of Diabetes Mellitus		
<2 Years	56	13.5
2–5 Years	109	26.3
6–10 Years	129	31.1
>10 Years	121	29.2
Follow-up Facilities		
No Routine Follow up	108	26.0
Primary Healthcare	69	16.6
Int Med/Endocrine Govt Hosp	196	47.2
Private Hospitals/Clinics	42	10.1
Pattern of Follow-up for Diabetes Care in Last Year		
Never Missed Follow-Up	187	45.1
Missed 1–2 Appointments	89	21.4
Missed > 2 Appointments	45	10.8
No Follow-up/Appointment Last Year	94	22.7
Diabetes control		
Uncontrolled	171	41.2
Controlled	244	58.8
Presence of Chronic Diseases		
Yes	217	52.3
No	198	47.7
Hypertension		
Yes	83	20.0
No	332	80.0
Thyroid Disorders		
Yes	47	11.3
No	368	88.7
Hyperlipidemia		
Yes	86	20.7
No	329	79.3
Chronic Kidney Disease		
Yes	18	4.3
No	397	95.7
Others Chronic Diseases		
Yes	26	6.3
No	389	93.7

T1DM: type 1 diabetes mellitus, T2DM: type 2 diabetes mellitus.

**Table 2:** Prevalence of missed insulin doses among patients with T1DM, T2DM, and those who were not aware of their type of DM.

	Overall (*N* = 415)	Patients with T1DM (*n* = 88)	Patients with T2DM (*n* = 206)	Patients Who Were Not Aware of Type of DM (*n* = 121)	*p* Values
Do you forget to take insulin?					<0.001
Yes	169 (40.7%)	51 (58%)	68 (33%)	50 (41.3%)	
No	246 (59.3%)	37 (42%)	138 (67%)	71 (58.7%)	
How many doses do you miss in a week?					0.012
I do not miss any dose	246 (59.3%)	37 (42%)	138 (67%)	71 (58.7%)	
1–2 doses	65 (15.7%)	22 (25%)	24 (11.7%)	19 (15.7%)	
3–4 doses	57 (13.7%)	17 (19.3%)	26 (12.6%)	14 (11.6%)	
5–6 doses	26 (6.3%)	8 (9.1%)	9 (4.4%)	9 (7.4%)	
Greater than 6 doses	21 (5.1%)	4 (4.5%)	9 (4.4%)	8 (6.6%)	

T1DM: type 1 diabetes mellitus, T2DM: type 2 diabetes mellitus, DM: diabetes mellitus.

**Table 3:** Self-reported reasons for nonadherence with insulin doses (*n* = 169).

Reasons for Missing Insulin Doses	Frequency (*n*)	Proportion (%)
Away from home	61	36.1
I cannot adhere to dietary regimen	41	24.3
Feeling embarrassed to take it in public	40	23.7
The time to take it is not appropriate	31	18.3
Forget	26	15.4
Took only when blood sugar is high	22	13.0
Time consuming	17	10.1
Regimen is complex	15	8.9
Fear of injection pain	7	4.1
Ran out of medication	7	4.1
Took only when felt sick	4	2.4

**Table 4:** Patient-reported factors to improve the adherence to insulin injections.

Factors	Frequency (*n*)	Proportion (%)
Minimize number of injections		
Yes	128	30.8
No	287	69.2
Convenient time regimen		
Yes	123	29.6
No	292	70.4
Confidence in taking medication in public		
Yes	93	22.4
No	322	77.6
Belief in efficacy of the treatment		
Yes	41	9.9
No	374	90.1
Social Support		
Yes	79	19.0
No	336	81.0

## Data Availability

Not applicable.
